# Long Non-Coding RNA Expression in B-Cell Precursor Acute Lymphoblastic Leukemia: Analysis of LINC-PINT, MEG3, BALR6, and ZEB1-AS1

**DOI:** 10.3390/life16071042

**Published:** 2026-06-23

**Authors:** Gabriel Mata Moreno, Edgar A. Turrubiartes Martínez, Lourdes Cecilia Correa González, Eduardo Roberto Caballero Lugo, Óscar Pérez Ramírez, Perla Niño Moreno, Esther Layseca Espinosa

**Affiliations:** 1 Medical Genomics Section, Research Center in Health Sciences and Biomedicine, Universidad Autónoma de San Luis Potosí, San Luis Potosi 78210, Mexico; gabriel.mata@uaslp.mx (G.M.M.); edgar.turrubiartes@uaslp.mx (E.A.T.M.); 2 Molecular and Translational Medicine Section, Research Center for Health Sciences and Biomedicine, Universidad Autónoma de San Luis Potosí, San Luis Potosi 78210, Mexico; 3 Hematology Laboratory, Faculty of Chemical Sciences, Universidad Autónoma de San Luis Potosí, San Luis Potosi 78210, Mexico; 4 Hospital Regional de Alta Especialidad “Dr. Ignacio Morones Prieto”, San Luis Potosi 78290, Mexico; cecilia.correa@uaslp.mx (L.C.C.G.); caballerolugo.hematopedia@gmail.com (E.R.C.L.); oscar.perez@uaslp.mx (Ó.P.R.); 5 Faculty of Chemical Sciences, Universidad Autónoma de San Luis Potosí, San Luis Potosi 78210, Mexico; 6 Department of Immunology, Faculty of Medicine, Universidad Autónoma de San Luis Potosí, San Luis Potosi 78210, Mexico

**Keywords:** lncRNA, leukemia, B-ALL, minimal residual disease

## Abstract

Background/Objectives: Long non-coding RNAs (lncRNAs) have been identified as potential biomarkers for cancer diagnosis and prognosis. In the present study, we proposed the analysis of four lncRNAs as a diagnostic support candidate for the follow-up of leukemia patients. The aim of this study was to characterize the expression of BALR6, LINC-PINT, MEG3, and ZEB1-AS1 in patients with B-cell acute lymphoblastic leukemia (B-ALL) at diagnosis and at the end of remission induction therapy. Methods: B-ALL diagnosis and MRD assessment were performed by flow cytometry, while lncRNA expression levels were quantified using TaqMan probe-based assays. Results: Fifteen pediatric patients with B-ALL were followed longitudinally. MRD evaluation identified seven refractory and eight remitted patients. Significant expression changes were observed for MEG3 in remitted patients and for BALR6 and LINC-PINT in refractory patients. No statistically significant differences were detected for ZEB1-AS1. Conclusions: Changes in MEG3, LINC-PINT, and BALR6 lncRNA expression are associated with treatment response and MRD status in pediatric B-ALL, supporting their potential role as complementary biomarkers to conventional MRD monitoring.

## 1. Introduction

Cancer is considered the seventh leading cause of death from non-communicable diseases among children under 5 years of age and the second among those aged 5 to 14 years. Leukemia is the most frequent clinical presentation, with a recorded incidence of 3.2 new cases and 1.3 deaths per 100,000 population [1]. In Mexico, the incidence reaches 5.76 new cases and 5.18 deaths per 100,000 population. Acute lymphoblastic leukemia accounts for 85.35% of total cases, of which 52.69% correspond to precursor B-cell acute lymphoblastic leukemia (B-ALL) [2,3,4].

Successful treatment of acute lymphoblastic leukemia relies largely on two critical factors: early diagnosis and accurate monitoring of minimal residual disease (MRD) [5]. MRD assessment has become a cornerstone of contemporary risk stratification, as numerous studies have consistently demonstrated its strong prognostic value for predicting relapse and guiding therapeutic decisions. However, in many low- and middle-income countries, access to MRD evaluation remains limited due to economic constraints and the restricted availability of specialized diagnostic assays [6]. These disparities in diagnostic capacity contribute to differences in clinical outcomes. Indeed, while the 5-year survival rate for pediatric acute lymphoblastic leukemia exceeds 85% in high-income countries, survival in Mexico remains approximately 60% [2,3,4,6].

Multiparametric flow cytometry is the gold standard for MRD diagnosis and evaluation. Antibody panels and analytical strategies have been refined and standardized in recent years to increase sensitivity and ensure reproducible, reliable results. Ideally, MRD assessment should be integrated into a comprehensive diagnostic approach that includes techniques such as fusion gene detection (via FISH or molecular biology) and karyotyping [7].

The main methodologies for MRD assessment—molecular techniques and flow cytometry—both exhibit high sensitivity and specificity [7]. However, molecular methods vary depending on the targeted genes and are limited by the low frequency of fusion gene alterations (approximately 42% of patients do not exhibit such changes) [8]. Meanwhile, flow cytometry is associated with elevated costs due to infrastructure and the technical expertise required for proper implementation. Therefore, there is a need to explore alternative approaches that offer MRD evaluation at a lower cost and greater accessibility. One promising option involves the use of long non-coding RNAs, which are being investigated as prognostic biomarkers in various types of cancer [9].

Long non-coding RNAs (lncRNAs) are RNA molecules longer than 200 nucleotides that lack protein-coding capacity but play critical regulatory roles in gene expression, including chromatin remodeling, transcriptional control, RNA processing, and translation. Dysregulated expression of lncRNAs has been strongly associated with the onset and progression of numerous malignancies [10]. Several lncRNA expression signatures have been proposed as biomarkers for a variety of cancers, including both solid tumors and hematologic malignancies [10]. Some of these signatures have been validated, further supporting the role of lncRNAs as valuable tools for cancer diagnosis, monitoring, and prognosis.

In B-cell acute lymphoblastic leukemia (B-ALL), lncRNAs have been shown to be directly involved in the initiation and progression of neoplastic processes via cancer hallmarks proposed by Di Gesualdo (2014) [10,11,12,13,14,15]. The lncRNAs evaluated in the present study were selected based on their reported involvement in biological processes associated with leukemogenesis. MEG3 functions as a tumor suppressor by promoting apoptosis and hematopoietic progenitor cell differentiation [16,17], thereby contributing to the regulation of cell survival and growth suppression. LINC-PINT participates in DNA damage repair and cell cycle control through modulation of the p53 signaling pathway and has been associated with apoptotic responses [18,19]. In contrast, BALR6 promotes leukemic cell proliferation and survival through activation of an SP1-driven transcriptional program [20], whereas ZEB1-AS1 contributes to leukemia maintenance by enhancing STAT3 signaling and other pathways related to cell proliferation and survival [21,22]. Collectively, these lncRNAs are linked to hallmark processes such as sustained proliferative signaling, evasion of growth suppressors, replicative immortality, and resistance to cell death, supporting their potential relevance as biomarkers of disease persistence and treatment response. Therefore, in the present study, we analyzed the relative expression of these lncRNAs and evaluated its association with MRD status as assessed by flow cytometry.

## 2. Materials and Methods

### 2.1. Ethics Statement and Bone Marrow Aspiration Samples

Samples were obtained under a protocol previously approved by the Research Ethics Committee from “Hospital Central Dr. Ignacio Morones Prieto” under registration CONBIOETICA-24-CEI-001-20160427. Bone marrow aspirate samples from patients with B-ALL were collected in EDTA tubes and kept at room temperature until processing (less than 24 h). Samples were collected at the time of diagnosis and at the end of remission treatment as part of routine laboratory tests.

### 2.2. Sample Dye and Flow Cytometry Acquisition

Twenty-five microliters of the bone marrow aspirate sample were lysed with 925 µL of FACS™ Lysing Solution 1× (BD Biosciences, San Jose, CA, USA; Cat. No. 349202) according to the manufacturer’s specifications. The resultant pellet was resuspended in 1 mL of phosphate-buffered saline. Cellular counting was performed using a hemocytometer and trypan blue. A volume of the sample containing 150,000 cells was incubated for 30 min with CD45 (Beckman Coulter, Marseille, France; Cat. No. A74765), CD19 (Beckman Coulter, Marseille, France; Cat. No. IM1284U), CD10 (BD Biosciences, San Jose, CA, USA; Cat. No. 340923), CD34 (BD Biosciences, San Jose, CA, USA; Cat. No. 348791), CD58 (Beckman Coulter, Marseille, France; Cat. No. IM1430), CD38 (BioLegend, San Diego, CA, USA; Cat. No. 356616) and CD81 (BioLegend, San Diego, CA, USA; Cat. No. 349508). Erythrocyte lysis and fixation were performed using BD FACS Lysing Solution, after which the sample was resuspended in a final PBS volume of 150 µL. Samples were acquired using a BD FACSCanto™ II flow cytometer (BD Biosciences, San Jose, CA, USA) until a minimum of 100,000 events were obtained. Analysis of the generated files was performed using Infinicyt software (version 2.0; Cytognos SL, Salamanca, Spain).

### 2.3. Sample Preparation and RNA Extraction

Bone marrow samples (600 µL) were processed for RNA extraction. Erythrocyte lysis was performed using Buffer EL (QIAGEN, Hilden, Germany; Cat. No. 79217); the resultant pellet was resuspended in 500 µL of NucleoZol^®^ (MACHEREY-NAGEL, Düren, Germany; Cat. No. 740404.200) and stored at −80 °C until RNA extraction. Extraction was performed according to the NucleoZOL protocol, and RNA was resuspended in 30 µL of nuclease-free water. Quality and concentration were assessed by nano spectrophotometry using an Epoch™ Microplate Spectrophotometer (BioTek Instruments, Winooski, VT, USA; Agilent Technologies, Santa Clara, CA, USA).

### 2.4. lncRNAs Evaluation

cDNA was synthesized from purified RNA using RevertAid Reverse Transcriptase (Thermo Fisher Scientific, Waltham, MA, USA; Cat. No. EP0441) with Oligo(dT) Primer (Thermo Fisher Scientific, Waltham, MA, USA; Cat. No. SO131) and RiboLock RNase Inhibitor (Thermo Fisher Scientific, Waltham, MA, USA; Cat. No. EO0382).

Quantitative PCR (qPCR) was performed using the following TaqMan™ Gene Expression Assays (20×): Hs05031477_s1 (LINC-PINT), FAM™-MGB probe (Thermo Fisher Scientific, Waltham, MA, USA; Cat. No. 4448484); Hs00292028_m1 (MEG3), FAM™-MGB probe (Thermo Fisher Scientific, Waltham, MA, USA; Cat. No. 4448484); Hs01398645_g1 (ZEB1-AS1), FAM™-MGB probe (Thermo Fisher Scientific, Waltham, MA, USA; Cat. No. 4426961); Hs00245445_m1, VIC™-MGB probe, 20× (Thermo Fisher Scientific, Waltham, MA, USA; Cat. No. 4448490); and a Custom TaqMan™ Gene Expression Assay (BALR6), FAM™-MGB probe, 20× (Thermo Fisher Scientific, Waltham, MA, USA; Custom Assay ID: APT2DV2). Reactions were carried out using TaqMan™ Universal PCR Master Mix (Advanced Master Mix Reagent) (Applied Biosystems, Foster City, CA, USA; Cat. No. 4444556) on a CFX96 Touch™ Real-Time PCR Detection System (Bio-Rad Laboratories, Hercules, CA, USA).

Data were analyzed using CFX Maestro™ Software v5.3.022.1030 (Bio-Rad Laboratories, Hercules, CA, USA). Relative lncRNA expression levels were calculated using the 2^−ΔΔCt^ method, with ABL1 serving as the endogenous control gene.

### 2.5. MRD Evaluation

**Figure 1:**
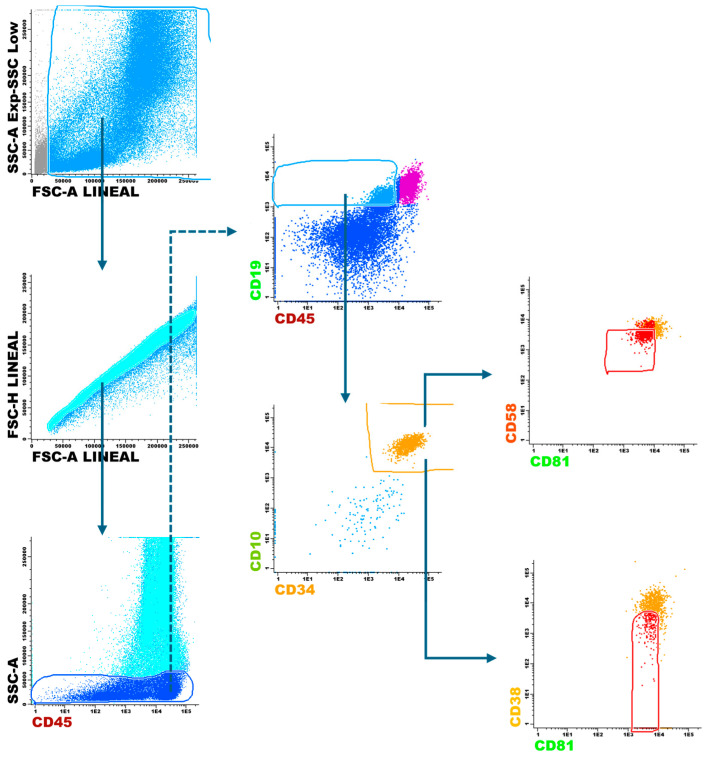
Gating strategy for MRD analysis. Cell populations were evaluated according to the initial leukemic immunophenotype (in this example by high CD10 and expression). B-cell precursor candidates were identified as low side-scatter events with variable CD45 expression and positivity for CD19. The leukemic population was subsequently refined based on CD10 and CD34 co-expression. Expression patterns of CD38, CD58, and CD81 were then used to discriminate hematogones (gold) from leukemic blasts (red) displaying a similar precursor B-cell phenotype, as previously described by Tsitsikov (2018) [23]. Blue arrows denote the sequential gating workflow employed during flow cytometric analysis, illustrating how each gated cell population was subsequently used to generate the next analytical plot.

A second bone marrow aspirate sample was taken at the end of the induction therapy. The sample was processed as previously described with the following modifications: at least 1,200,000 cells were incubated with antibodies, and then 1,000,000 events were acquired in the cytometer. MRD was analyzed using the strategy shown in Figure 1 to distinguish neoplastic cells from hematogones. The cutoff was established at 0.01% of the total acquired events. Patients were considered MRD-positive when at least 100 cells exhibiting the leukemia-associated immunophenotype identified at diagnosis were detected, whereas patients with fewer than 100 such cells were considered MRD-negative.

### 2.6. Statistical Analysis

Paired comparisons of mean relative gene expression levels between diagnosis and MRD evaluation were performed using the Wilcoxon matched pairs signed-rank test (two-tailed). Comparisons of gene expression levels between remitted and refractory patients were performed at diagnosis and MRD evaluation using the Mann–Whitney U test (two-tailed), given the independent nature of the study groups. All statistical analyses were conducted using GraphPad Prism (version 10.4.1; GraphPad Software, San Diego, CA, USA). A *p*-value < 0.05 was considered statistically significant.

## 3. Results

### 3.1. Cohort Characteristics

Complete follow-up data was obtained for 15 patients diagnosed with B-ALL, from the time of diagnosis through MRD evaluation at the end of remission induction therapy. The mean age of the patients was 9.4 (±4.5) years, and males accounted for 80% of the cases. Clinical features of patients are summarized in Table 1; detailed sociodemographic data are included in Table S1.

### 3.2. MRD Evaluation at the End of Induction Therapy

MRD assessment by flow cytometry identified seven patients with positive MRD, classified as refractory disease patients, and eight patients who achieved remission, classified as remitted disease patients. Details are shown in Table 1.

### 3.3. lncRNA Expression Profiles According to Treatment Response

The relative expression of selected long non-coding RNAs (lncRNAs) was evaluated at diagnosis and at the end of induction therapy and then stratified according to treatment response (MRD+ vs. MRD−). Details are shown in Table 2.

#### 3.3.1. Expression Levels of lncRNAs at Diagnosis and at the End of Induction Therapy

**Figure 2:**
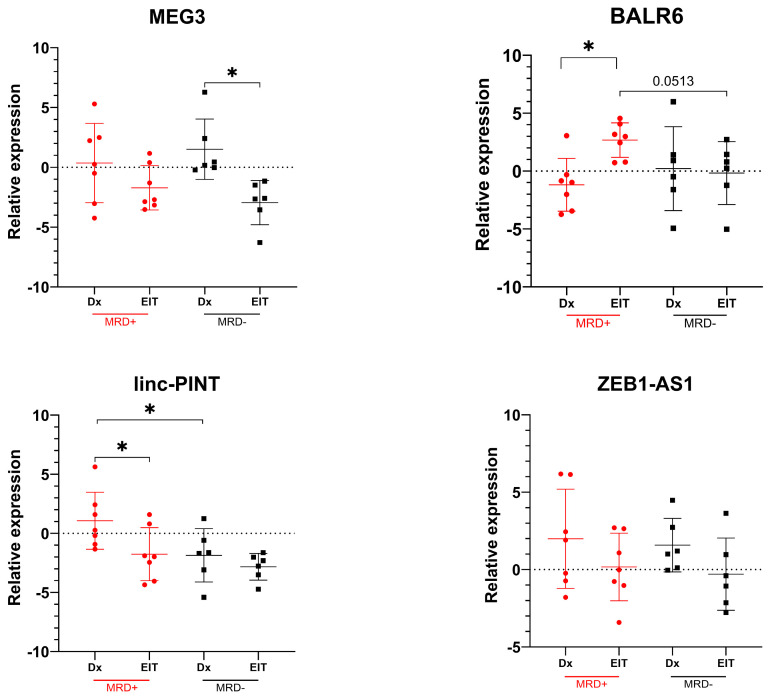
Relative lncRNA expression levels at diagnosis (Dx) and at the end of remission induction therapy (EIT). Patients with refractory disease (MRD-positive) are shown in red, whereas patients who achieved remission (MRD-negative) are shown in black. An asterisk (*) denotes a statistically significant difference (*p* < 0.05).

At diagnosis, a statistically significant difference in LINC-PINT expression was observed, with higher expression levels in patients with treatment-refractory disease compared to those who achieved remission. Furthermore, when evaluating the expression of the molecules of interest at the end of induction therapy, a trend toward increased BALR6 expression was detected in patients with refractory disease relative to the remission group (*p* = 0.0513). Details are shown in Figure 2.

#### 3.3.2. Differences in lncRNA Expression from Diagnosis to End of Induction Therapy

Regarding treatment-associated changes following induction therapy, a statistically significant increase in BALR6 expression was observed in patients with treatment-refractory disease. In contrast, MEG3 expression showed a statistically significant decrease in patients who achieved remission. Additionally, a significant reduction in LINC-PINT expression was detected in patients who did not achieve remission. No statistically significant differences were observed in ZEB1-AS1 expression when comparing baseline and end-of-treatment levels in either group. Details are shown in Figure 2.

## 4. Discussion

The natural history of leukemia includes the possibility that treatment-resistant clones may emerge during pharmacological therapy, leading to treatment failure or disease relapse after therapy completion. Such events may occur even several years after the conclusion of induction therapy aimed at achieving remission. To reduce this risk, strategies such as the assessment of MRD—primarily through flow cytometry and, in certain cases, through molecular methods—have been developed to evaluate the likelihood of relapse in individual patients (van Dongen, van der Velden et al., 2015) [7]. However, these methodologies present an inherent limitation related to the resolution capacity of the available technologies; under no circumstances can it be guaranteed that a patient is entirely free of cells exhibiting neoplastic processes or characteristics (van Dongen, van der Velden et al., 2015; Li, 2022) [8,24].

Consequently, there is a clear need to identify novel approaches capable of assessing patient status in a more comprehensive manner. These approaches should not rely solely on the expression of markers associated with active neoplastic processes but rather on indicators that reflect the intrinsic cellular capacity to initiate or sustain malignant transformation.

In the present study, we sought to characterize this capacity within bone marrow cells by evaluating five principal oncogenic hallmarks: sustained proliferative signaling, evasion of growth suppressors, activation of invasion and metastasis processes, enabling of replicative immortality, and resistance to cell death (Schmitt and Chang 2016) [25]. The long non-coding RNAs (lncRNAs) analyzed were selected based on existing evidence of their involvement in these processes, and their expression profiles were examined in relation to disease remission status (MRD-positive versus MRD-negative).

The significantly higher levels of MEG3 expression observed in patients who achieved remission suggest the ability of these cells to regulate apoptotic pathways through p53 signaling and other non-canonical mechanisms [26]. As previously reported by Gao (2021) [27] and Zhang and Qin (2024) [17], this regulatory capacity translates into a more favorable prognosis and an enhanced potential of hematopoietic precursor cells to undergo appropriate differentiation. Interestingly, despite the association between elevated MEG3 expression and remission, a reduction in MEG3 levels was observed following induction therapy. We propose that this finding may reflect changes in the cellular composition of the bone marrow during treatment response. As induction therapy reduces the leukemic burden and promotes marrow recovery, the overall MEG3 expression profile may shift due to the progressive replacement of leukemic cells by regenerating normal hematopoietic populations.

Although studies by Wang, Guo et al. (2021) [19] and Lin, Chen et al. (2024) [28] have reported that elevated expression levels of LINC-PINT in solid tumors are associated with favorable clinical outcomes, our results reveal a markedly different scenario. The present study found that high LINC-PINT expression at the time of diagnosis was associated with a poorer response to induction therapy. This is likely to be due to the enhanced ability conferred by this lncRNA to repair DNA damage, as previously described by Wang, Guo et al. (2021) [19]. While these earlier studies focused on solid malignancies, Garitano-Trojaola, San José-Enériz et al. (2018) [29] demonstrated that, under basal conditions, B-lineage cells (CD19^+^) from healthy individuals exhibit low LINC-PINT expression, which is consistent with our observations in patients who achieved remission. Therefore, high levels of LINC-PINT expressions appear to enhance cellular DNA repair mechanisms, thereby mitigating the cytotoxic effects of administered therapies.

In agreement with the findings reported by Rodríguez-Malavé, Fernando et al. (2015) [30], our results demonstrate that high expression of the lncRNA BALR6 promotes the proliferation of leukemic clones, as evidenced by increased expression levels in MRD-positive patients at the end of induction therapy. These findings provide in vivo validation of previously reported experimental data. Additionally, patients who achieved remission following induction therapy exhibited low BALR6 expression levels at diagnosis and maintained comparable levels at treatment completion.

Regarding ZEB1-AS1, contrary to the observations reported by Wang, Du et al. (2017) [31], our data did not reveal statistically significant differences between patient groups or MRD stages. According to Hu, Cesano et al. (1993) [32], leukemic B cells display limited responsiveness to IL-11, either alone or in combination with other cytokines, which may account for the absence of detectable effects.

While these findings suggest a potential association between lncRNA expression and treatment response, they should be interpreted in the context of the limited cohort size. Therefore, the observed trends require confirmation in larger studies before definitive conclusions regarding their clinical utility can be drawn.

It is important to highlight that the biological roles of lncRNAs may differ substantially between solid and hematological malignancies. For example, several studies have reported that elevated expression of lncRNAs such as MEG3 is associated with favorable clinical outcomes and tumor-suppressive functions in solid cancers [17]. But our findings suggest a more complex pattern in pediatric B-ALL, showing dynamic changes following induction therapy as described by Wang, Airong et al. (2022) [33]. These discrepancies may reflect that differences exist between solid and hematologic malignancies. For these reasons, the functional role of lncRNAs cannot be generalized across malignancies and should be interpreted according to the biological context of each cancer type.

In context, a limited number of lncRNA-validated panels with diagnostic and prognostic relevance have already been reported for various malignancies, including gallbladder carcinoma (Mishra, Srivastava et al., 2024) [34], esophageal squamous cell carcinoma (Tong, Wang et al., 2015) [35], lung squamous cell carcinoma (Hu, Jie et al., 2016; Satpathy, Krug et al., 2021) [36,37], hepatocellular carcinoma (Li, Shi et al., 2019; Zeng, Guo et al., 2019) [38,39], breast cancer (Li, Wang et al., 2018; Shaath, Elango et al., 2021) [40,41], colorectal cancer (Matouk, Abbasi et al., 2009) [42], clear cell renal cell carcinoma (Wu, Wang et al., 2016; Zeng, Lu et al., 2019) [43,44], head and neck squamous cell carcinoma (Xing, Zhang et al., 2019; Wang, Bian et al., 2021) [45,46], and acute myeloid leukemia (Wang, Tian et al., 2018; Chen, Wang et al., 2019) [47,48], among others.

## 5. Conclusions

Overall, this study represents, to the best of our knowledge, the first evaluation of MEG3, LINC-PINT, BALR6, and ZEB1-AS1 expression in a Mexican pediatric B-ALL cohort and the first assessment of their expression dynamics between diagnosis and MRD evaluation. Our findings demonstrate that cellular processes associated with leukemic persistence can be indirectly monitored through the evaluation of these molecules by providing a more comprehensive view of the residual cellular population persisting after induction therapy, contributing to improved risk stratification and the development of more personalized disease-monitoring and therapeutic decision-making strategies. Nevertheless, larger multicenter studies are required to validate these findings and establish the clinical utility of lncRNA-based biomarkers in routine leukemia monitoring.

## Figures and Tables

**Table 1:** Clinical features of B-ALL pediatric patients enrolled in this study.

Patient_ID	1	2	3	4	5	6	7	8	9	10	11	12	13	14	15
Sex (F/M)	M	M	F	M	M	M	M	M	M	M	F	M	F	M	M
Age (years)	6	10	5	15	2	13	13	5	4	9	14	14	12	9	8
Diagnosis (EGIL subtype)	II	II	II	II	ND	ND	II	II	II	II	III	II	II	II	II
Leukocytes (K/μL)	3.86	2.30	6.49	5.98	4.49	21.20	24.43	1.68	0.76	3.38	1.44	7.41	3.29	ND	ND
MRD (%)	<0.01%	<0.01%	<0.01%	0.0114	<0.01%	0.439	0.04	<0.01%	0.028	0.017	<0.01%	<0.01%	0.0118	0.09	0.017

Abbreviations: ND—no data. EGIL—European Group for the Immunological Characterization of Leukemias.

**Table 2:** Relative lncRNA expression levels at diagnosis and at MRD evaluation.

	Refractory Disease (MRD-Positive)								Remitted Disease (MRD-Negative)							
	BALR6		LINC-PINT		MEG3		ZEB1-AS1		BALR6		LINC-PINT		MEG3		ZEB1-AS1	
	Dx	MRD	Dx	MRD	Dx	MRD	Dx	MRD	Dx	MRD	Dx	MRD	Dx	MRD	Dx	MRD
Mean	−1.19	2.67	1.07	−1.76	0.36	−1.71	1.99	0.17	0.21	−0.18	−1.86	−2.83	1.51	−2.95	1.58	−0.30
SD	2.28	1.49	2.41	2.25	3.31	1.85	3.21	2.18	3.62	2.71	2.26	1.13	2.52	1.85	1.73	2.33
*p*-value	**0.0469**		**0.0312**		0.1562		0.5781		0.8438		0.5625		**0.0312**		0.2188	

**Abbreviations:** Dx, diagnosis; MRD, minimal residual disease at the end of remission induction treatment. Bold values indicate statistically significant differences (*p* < 0.05).

## Data Availability

Data are contained within the article and Supplementary Materials.

## Supplementary Materials

The following supporting information can be downloaded at: https://www.mdpi.com/article/10.3390/life16071042/s1, Table S1: Sociodemographic and clinical data.

## Abbreviations

The following abbreviations are used in this manuscript:

B-ALL	B-cell acute lymphoblastic leukemia
lncRNA	Long non-coding RNA
MRD	Minimal residual disease
CD	Cluster of Differentiation
